# Supplementary material for: Sulfur and chlorine budgets control the ore fertility of arc magmas
Source: Nat Commun. 2022 Jul 21;13:4218. doi: 10.1038/s41467-022-31894-0 (PMC9304346; doi:10.1038/s41467-022-31894-0)
Supplement: Supplementary file 1 — Supplementary Information [file 41467_2022_31894_MOESM1_ESM.pdf]

Sulfur and chlorine budgets control the ore fertility of arc magmas

Carter Grondahl and Zoltán Zajacz

SUPPLEMENTARY INFORMATION

to article in *Nature Communications*

SUPPLEMENTARY DISCUSSION

Linking the modern arc to past porphyry Cu ore genesis

A detailed study in the vicinity of the giant, ~5 Ma El Teniente porphyry Cu-Mo deposit documented the local magmatic evolution from Oligocene to Pliocene[1]. Through time, magmatism in the El Teniente region underwent a geochemical evolution from low- to high-K, tholeiitic to calc alkaline, increasing light REE / heavy REE ratios, progressive Sr-Nd-Pb isotopic enrichment, and increasingly hydrous phenocryst assemblages. Ref. [1] also demonstrates the close resemblance of this temporal trend to the spatial trend observed through the modern SVZ from south to north. Although usually proposed to originate in the mantle or lower crust, the source of this geochemical trend remains controversial, with explanations including lower crustal mixing, assimilation, storage, and hybridization (MASH) of mantle-derived magma[2], equilibration with locally variable lithosphere[3], contamination of the mantle wedge by subduction-related erosion and entrainment of continental crust from the western margin of the South American plate[1,4,5], contamination of the mantle wedge by subcontinental lithospheric mantle entrained in mantle corner flow[6], and a dominant influence from subducting plate-derived melts or fluids[7].

There is also an important tectonic contrast between the north and south[8]. North of ~37 °S, the stress regime is compressional and favours the development of reverse faults with which the major volcanoes are spatially associated. In the south, the dextral transpressional stress regime is expressed as the

Loquiñe-Ofqui Fault Zone, and in this section of the arc both major and minor volcanoes (with locally variable compositions)[9] are typically associated with the major and minor faults of this dominant tectonic feature. Like the geochemistry, this tectonic contrast is also mirrored in the El Teniente-area record wherein a non-contractional Miocene regime associated with relatively thin crust eventually evolves into a contractional Pliocene regime with crustal thickening and periodic eastward arc-front migration[1].

The geochemical and tectonic evidence shows that ore-forming SVZ magmas are associated with arc segments that evolve to compressional stress regimes and thickened crust, which promote prolonged magma storage, differentiation, and homogenization prior to ascent. Sampling volcanoes along the modern SVZ therefore yields insight into long-term trends in arc magmatism which culminate in world-class porphyry Cu deposit formation.

Assimilation and degassing in the south

Key evidence showing that degassing occurs at depths greater than the upper crust includes the coupled decrease in S and Cl as differentiation proceeds, best exemplified by Villarrica and Planchon in Fig. 3. Importantly, highly incompatible elements like K_2O exhibit large increases in concentration (>2-3x) that are not accompanied by a complementary range in major element concentrations permissive of extensive crystal fractionation as the sole explanation (Fig. 3 and Supplementary Figs. 4 and 5). This observation can be explained by the assimilation of low-degree crustal melts, and thermal constraints restrict this process to deeper crustal levels.

Contamination of mafic SVZ magma as evidenced by incompatible trace element ratios and time-sensitive indicators including U-series disequilibrium and $^{10}Be/^{9}Be$ (e.g., refs. [9,10]) further demonstrate the potential for a locally variable balance of mantle and crustal influences to raise large-ion lithophile

element (LILE) concentrations. One likely contaminant source is the residual plutonic material left behind during differentiation and ascent of previous magma batches, which have been shown to interact with some ascending southern SVZ magmas[10]. It is likely that these somewhat older but broadly co-genetic magmas lost a significant fraction of their S and Cl budget during solidification prior to contaminating modern SVZ magma either by separation and loss of late-stage fractional melts and/or by crystallization-induced degassing. Therefore, the partial melts of these rocks assimilated by the modern magmas in newly developed lower-crustal hot zones will be S- and Cl-poor. If this contaminant is genetically similar to the modern magma, such as an ancestral plutonic 'root', it will be difficult to distinguish by traditional Sr-Nd-Pb isotope ratios[2,9-11].

SUPPLEMENTARY TABLES

Supplementary Table 1 | Criteria for pSMI filtering, based on host Mg# and SMI K_2O concentration, both of which must be satisfied to be considered a pSMI.

Volcano	LA-ICP-MS		EPMA	
	Mg#	K_2O	Mg#	K_2O
San Jose	≥80	≤1.8	≥78	≤2.2
Maipo	≥80	≤1.8	-- [a]	--
Planchon	≥77	≤1.2	≥75 [b]	≤1.0
Los Hornitos	≥84	≤1.0	≥81	≤1.0
Antuco	≥80	≤0.8	≥79	≤0.8
Llaima	≥74	≤0.8	≥74	≤1.0
Villarrica	≥81	≤0.6	≥76 [c]	≤0.72

a. All of the most primitive re-heated SMI (SiO_2 ≤~61 wt%; n=5) included; See *Methods*.
b. Four EPMA included w/ <1 K_2O but Mg#=69,70,72,74 because their S-Cl matches the other pSMI from higher Mg# (n=9).
c. Lower Mg# filter for EPMA because no SMI were permissive of EPMA analysis in the sample with contributed most of the LA-ICP-MS pSMIs (sample ZZVL).

Supplementary Table 2 | The occurrence of magmatic sulfide phases in the studied samples

Volcano	# of samples	Sulfides?	# per sample section	Host mineral	Type	Size (μm)
San Jose	1	Yes	≤1	Olivine, Fe-oxide	MSS	10 - 20
Maipo	3	Yes	≤1	Pyroxene	MSS	5 - 25
Planchon	3	No	--	--	--	--
Los Hornitos	2[a]	No	--	--	--	--
Antuco	4	No	--	--	--	--
Llaima	3	No	--	--	--	--
Villarrica	3	No	--	--	--	--

a. One sample was a poorly consolidated coarse lithic tuff, and prepared as mineral separates.

Supplementary Table 3 | Sulfide fractionation model results

Total S loss	S^{2-} (ppm)	1,300 *(720)*[a]	Solid : Liquid sulfide		0.97 : 0.03	
	MSS (wt%)	0.36 *(0.20)*				
						Northern pSMI
Proportion remaining (%)	Cu	35 *(19)*	Concentration remaining in melt	Cu (ppm)	56 *(31)*	56 *(10)*
	Au	31 *(14)*		Au (ppb)	1.7 *(0.8)*	1.8 *(1.1)*
	Ag	89 *(52)*		Ag (ppb)	88 *(51)*	54 *(23)*
Proportion lost (%)	Cu	65 *(36)*	Concentration in hypothetical bulk sulfide	Cu (wt%)	2.9 *(1.6)*	
	Au	69 *(30)*		Au (ppm)	1.1 *(0.5)*	
	Ag	11 *(6)*		Ag (ppm)	3.0 *(1.7)*	

a. Italicized values in parentheses are absolute 1σ uncertainties as described in the text for S loss, and derived from partition coefficient uncertainties provided by ref. [12] for Cu, Au and Ag concentrations. For Northern pSMI they represent 1σ uncertainties from the pSMI dataset.

Supplementary Table 4 | Typical LA-ICP-MS instrumental conditions

ICP-MS		LA	
Ar carrier gas	0.9-0.93 L/min	He carrier gas	1 L/min
U and Th sensitivity[a]	4-5 x 10^6 cps	Repetition rate	15 Hz
U/Th	~1	Energy density on sample surface	5-7 J/cm^2
ThO/Th	<0.3%		
^{21}X/ ^{42}Ca	<0.3%	Spot shape/size	SMI/Hosts: Variable<br>Standards: Variable

Standards[b]	Elements
NIST 610	Ag, Au, Pt
In-house S-rich andesite glass	S
GSD-1g	All others

Dwell times	Elements
5 ms	^{23}Na, ^{25}Mg, ^{27}Al, ^{29}Si, ^{31}P, ^{39}K, ^{44}Ca, ^{47}Ti, ^{51}V, ^{53}Cr, ^{55}Mn, ^{56}Fe, ^{85}Rb, ^{88}Sr, ^{93}Nb ^{137}Ba, ^{140}Ce, ^{208}Pb
10 ms	^{9}Be, ^{11}B, ^{45}Sc, ^{65}Cu, ^{89}Y, ^{91}Zr, ^{95}Mo, ^{172}Yb, ^{178}Hf, ^{181}Ta, ^{232}Th, ^{238}U
30 ms	^{34}S, ^{107}Ag
100 ms	^{195}Pt
120 ms	^{197}Au

a. Measured on NIST 610 with a 40 μm beam size, 10 Hz repetition rate, and 6 J/cm^2 energy density on the sample surface
b. GSD-1g is a USGS standard with a basaltic matrix and ~40 ppm of most trace elements[13]. Because Au and Pt are poorly characterised in GSD-1g, and because this glass contains platinum group element nuggets, Ag, Au and Pt concentrations were determined using NIST 610 within which these elements are better constrained especially for laser beam diameters ≥40 μm[14]. The S standard contains 2,890 ppm S and was synthesized in-house for the purpose of S analysis by LA-ICP-MS.

Supplementary Table 5 | Mass interferences and corrections during LA-ICP-MS analyses

Mass	Interference	Mineral	Production rate[a]	Contribution[b]
^{107}Ag	$^{91}Zr^{16}O$	Zircon	<0.03%	15-20%
^{195}Pt	$^{179}Hf^{16}O$	Zircon	<0.02%	20-30%
^{197}Au	$^{181}Ta^{16}O$	Tantalite	<0.02%	5-10%

a. Production rate = [^{107}Ag signal in zircon] cps / [^{91}Zr signal in zircon] cps.

b. Typical amount of the measured signal attributed to mass interference and subsequently subtracted.

Supplementary Table 6 | Mantle melting model parameters

	Olivine	Ortho-pyroxene	Clino-pyroxene	Spinel	Garnet	Sulfide[b]	MSS	SL	Initial Concentration[c]
Solid[a]	0.5	0.25	0.15	0.05	0.05	0.0006			
Melt	-0.1	0.25	0.61	0.04	0.2	0.0036			
D Cu	0.12	0.09	0.13	0.25	0.03	331	280 *(120)*	790 *(270)*	25 ppm *(10)*[d]
D Mo	0.05	0.035	0.02	0.01	0.01	61.7	66 *(7)*	23 *(2)*	36 ppb *(20)*
D Ag	0.01	0.01	0.01	0.01	0.01	63	20 *(8)*	450 *(190)*	6 ppb *(4.2)*
D Au	0.01	0.01	0.01	0.01	0.01	745	150 *(40)*	6,100 *(2100)*	1.3 ppb *(0.7)*
D Pt	0.01	0.01	0.01	0.01	0.01	18,415	4,350	145,000	6.9 ppb *(2.3)*

a. Solid and melt mineral modes are from ref. [15], except for sulfide which corresponds to 220 ppm S in the mantle and 1,300 ppm S^{2-} solubility in the mantle melt (ref. [16]).

b. Sulfide partition coefficients (D-values) are based on an MSS-SL ratio of 9:1 that was determined by using the best fit of modeled Pt and Au concentrations to those measured in pSMI.

c. Initial concentrations are an average of primitive (ref. [17]) and depleted (ref. [18]) mantle.

d. Italicized numbers are absolute uncertainties propagated from errors provided for primitive and depleted mantle estimates.

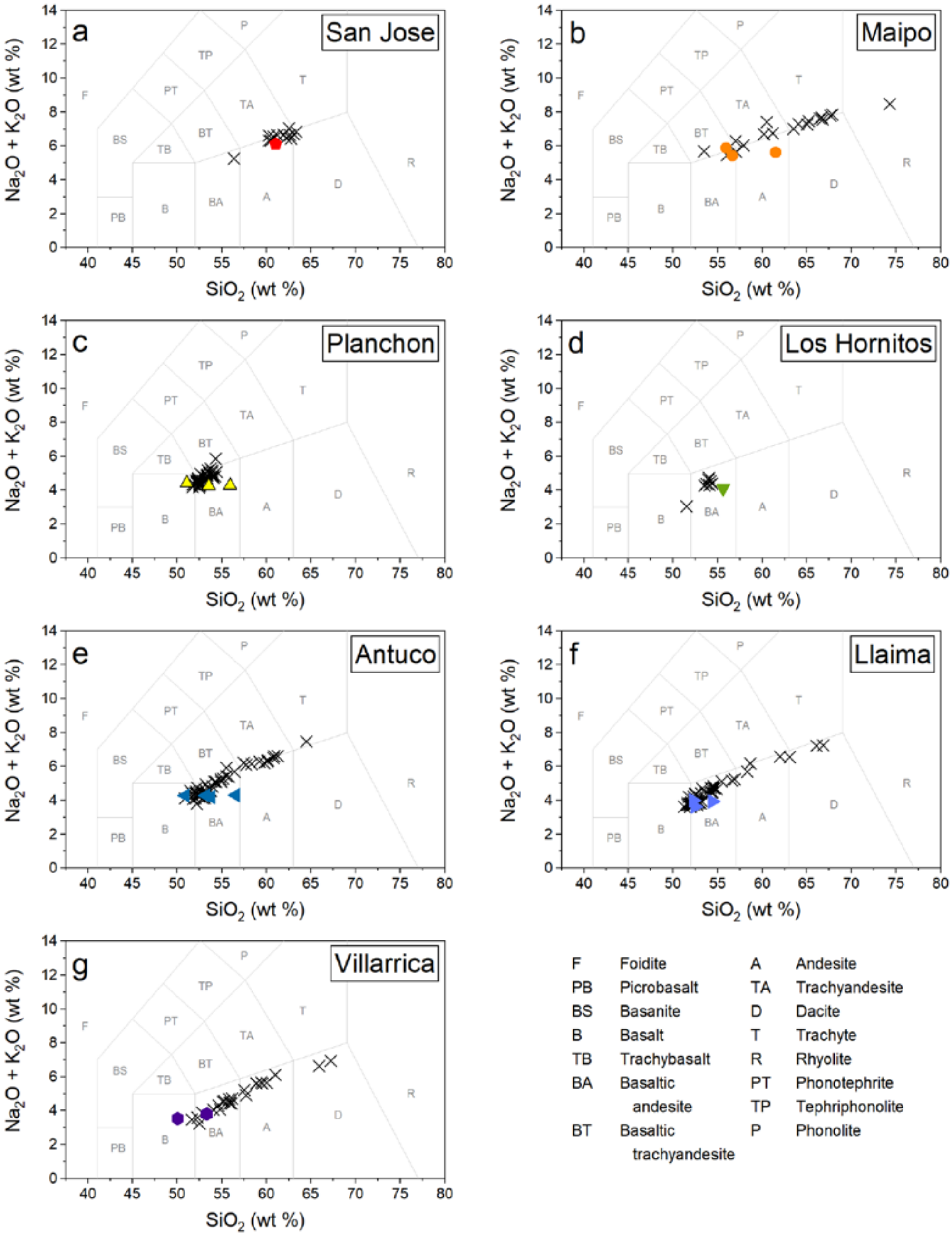

Supplementary Fig. 1 | The whole rock composition of the studied samples in the comparison to reported rock compositional ranges from each volcano. Coloured symbols are from this study, and crosses are literature values for: San Jose[3]; Maipo[19]; Planchon[20]; Los Hornitos[21,22]; Antuco[23]; Llaima[24]; and Villarrica[25]. Full compositions are given in the Supplementary Data.

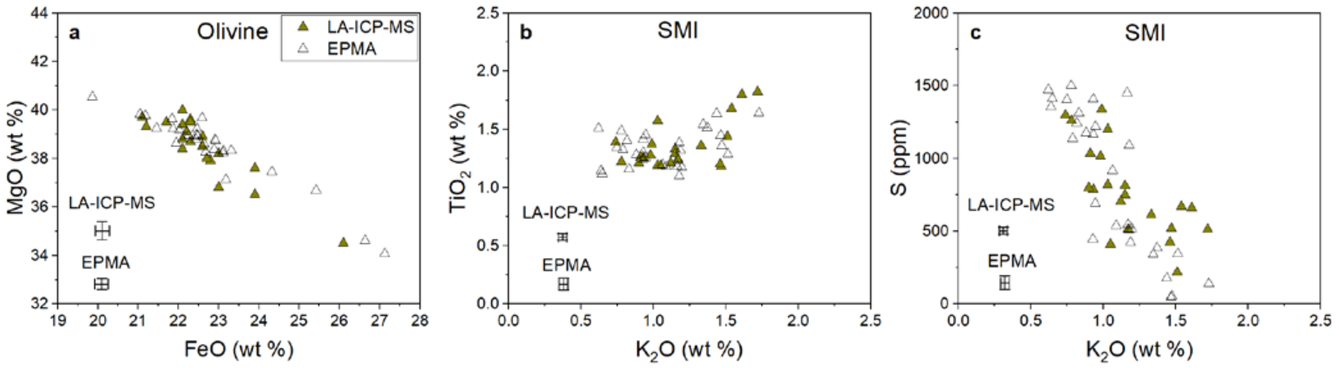

Supplementary Fig. 2 | Comparison of LA-ICP-MS and EPMA measurements from Planchon. Crosses show representative 1σ uncertainties for each method in the lower left. The two methods show good agreement and yield the same population of compositions for both the host (**a**) and the SMI (**b**, **c**).

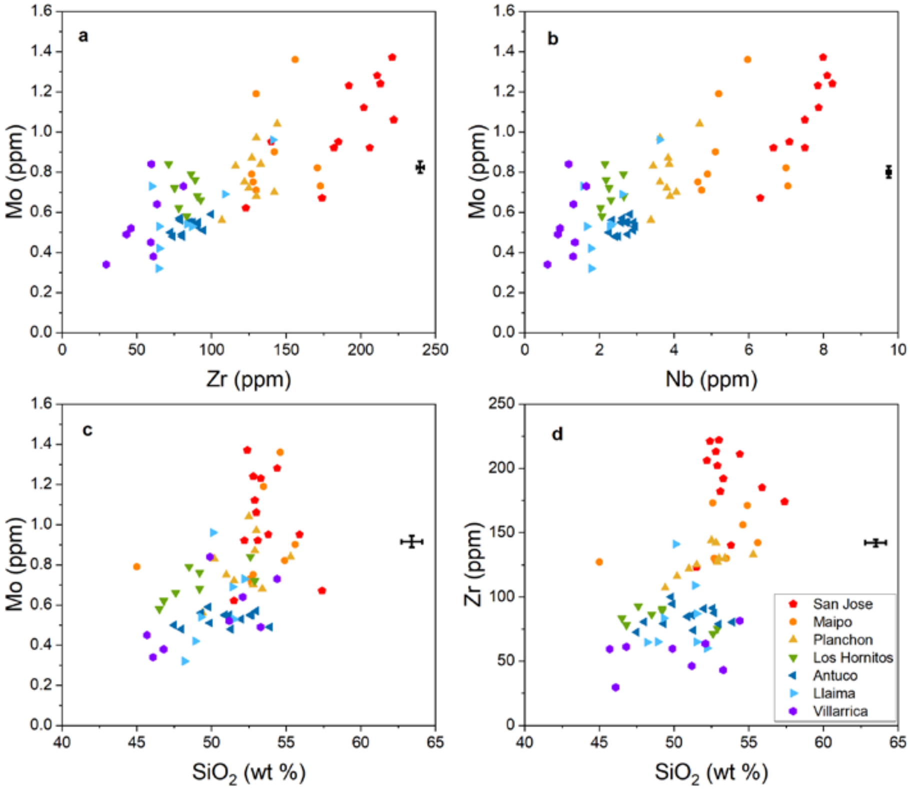

Supplementary Fig. 3 | The pSMI concentrations of Mo as a function of other incompatible fluid-immobile trace elements trace elements (a, Zr; b, Nb), and Mo and Zr variation with SiO_2 (c, d). Crosses along the right margin of each plot show representative 1σ uncertainties. Simple batch melting calculations with appropriate extents of melting (6% in the north and 12% in the south)[26] and assuming incompatible Mo behaviour in mantle minerals can reproduce the Mo concentrations of both the northern (~1 ± 0.25 ppm) and southern (~0.5 ± 0.25 ppm) pSMI by invoking a concentration in the mantle (0.065 ± 0.015 ppm) that is higher but within error of both the primitive (0.047 ± 0.019 ppm)[17] and averaged primitive-depleted (0.036 ± 0.020 ppm)[17,18] mantle. However, the much higher pSMI Mo concentrations in the north than the south for a given SiO_2 concentration (**c**) are consistent with increased Mo addition during magma differentiation in the lower crust (e.g., ref. [27]). Although it is difficult to determine whether the Mo-rich component is incorporated during any or all of lower crustal contamination (e.g., ref. [2]), subduction erosion (e.g., ref. [5]) or interaction with metasomatized subcontinental lithospheric mantle (e.g., ref. [6]), MASH-type processes will always favor Mo ore-fertility in the likely case that the lower crust contributes Mo.

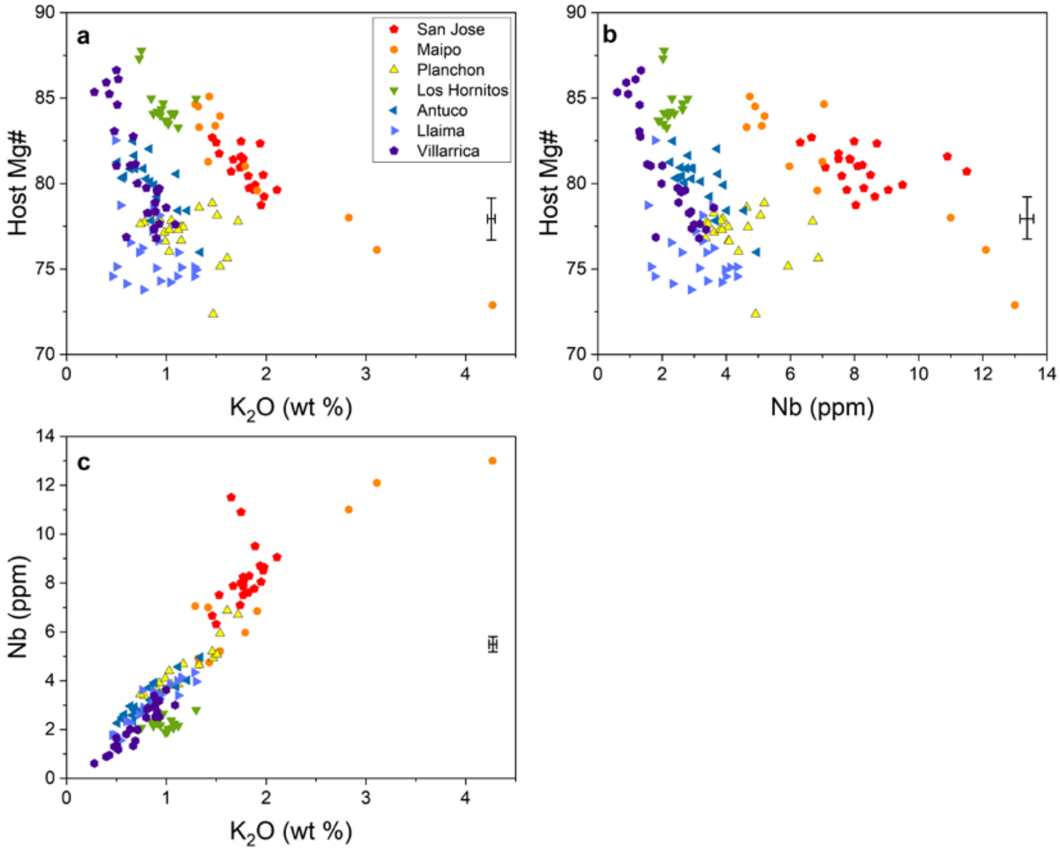

Supplementary Fig. 4 | Variation in fSMI of incompatible elements that are nominally fluid mobile (K_2O) and immobile (Nb) during subduction, and host mineral Mg#. Crosses along the right margin show representative 1σ uncertainties. As differentiation proceeds (including crystallization and assimilation) and Mg# decreases, incompatible elements (**a**, **b**) increase more significantly at Maipo and San Jose than to the south indicating increased assimilation of crustal melts. Overall, the large range in the concentration of highly incompatible elements over a narrow range in host Mg#, and while the magma remained in the olivine stability field for most volcanoes, demonstrates the significance of magma contamination by assimilated low-degree crustal melts. The significant span of highly incompatible trace element concentrations at a fixed host Mg# as apparent at several volcanoes reflects the buffering of major element concentrations and Fe/Mg ratios by a crystal mush at the site of crustal assimilation, likely at lower to mid crustal depths.

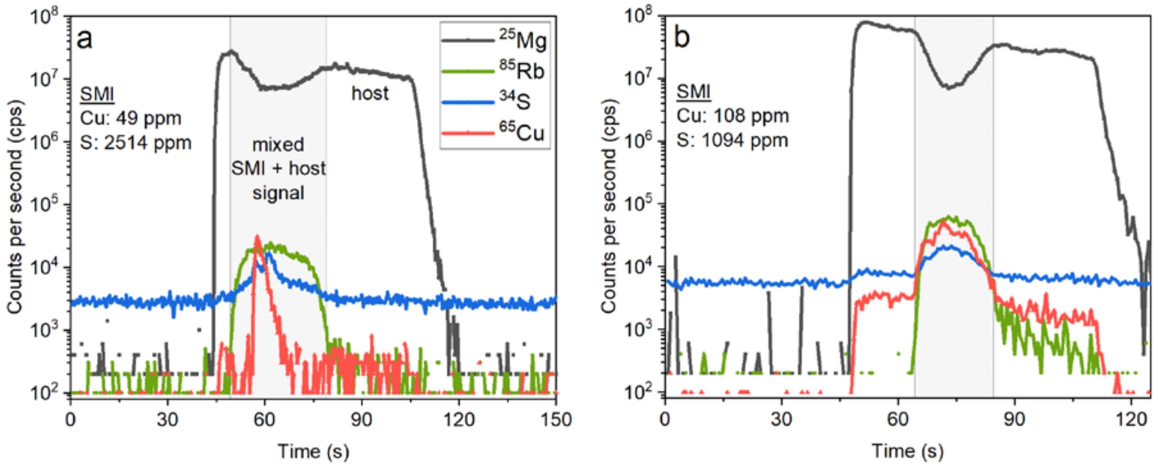

Supplementary Fig. 5 | Example LA-ICP-MS analyses from San Jose (a) and Antuco (b). Analyses from San Jose and Maipo typically exhibit strong, localized S-peaks (**a**), whereas analyses from the other volcanoes typically have S distributed more evenly throughout the SMI signal interval (**b**). Copper is typically correlated with the S peak when present.

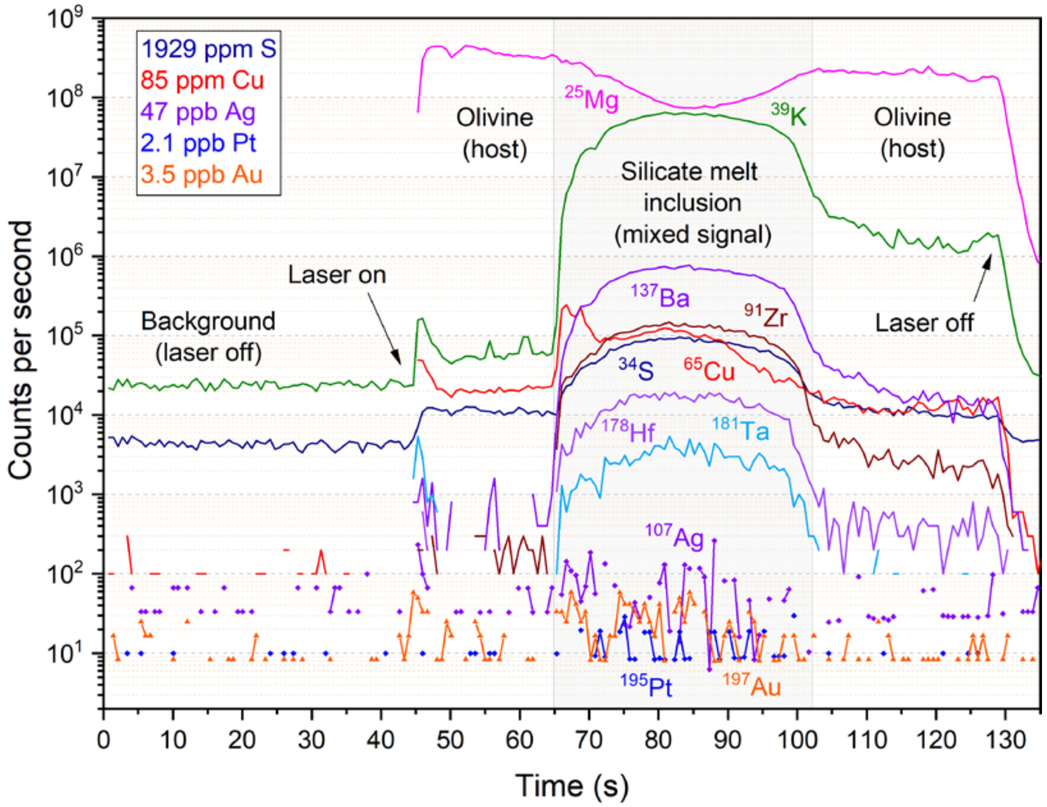

Supplementary Fig. 6 | Example LA-ICP-MS analysis of a large (72 x 95 µm) SMI from Los Hornitos. Interference-corrected count rates are shown for ^{107}Ag, ^{195}Pt, and ^{197}Au. The signals of these elements within the silicate melt inclusion interval are clearly distinguishable from those in the background and host mineral intervals. The corresponding concentrations in the final quantified SMI composition are shown in the top left.

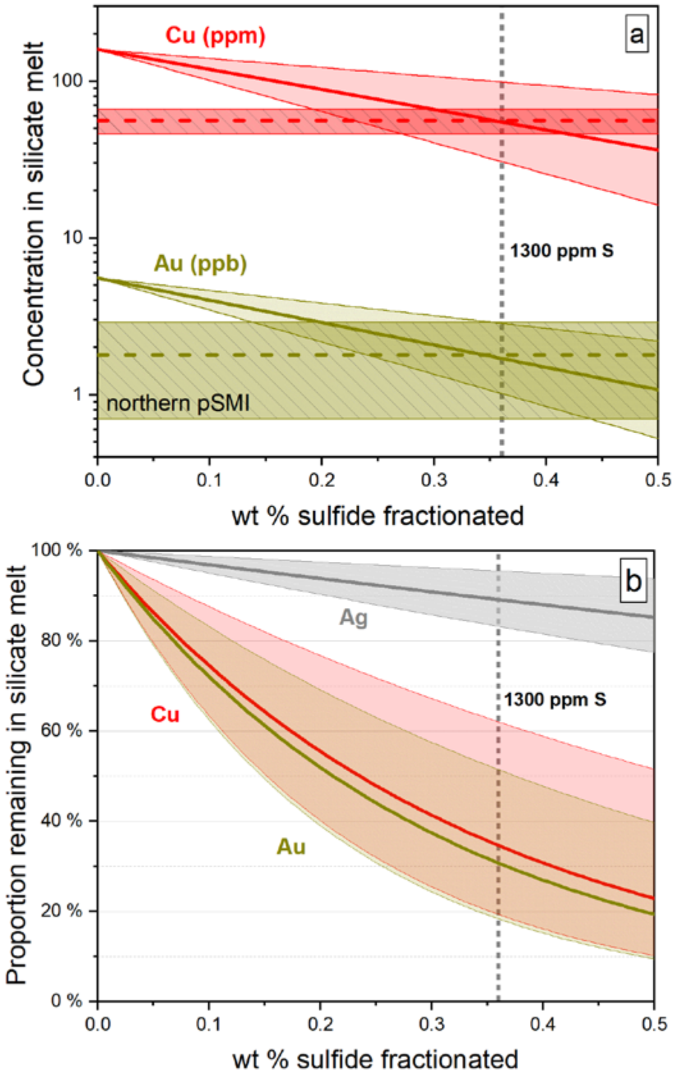

Supplementary Fig. 7 | Modeled ore metal scavenging during fractionation of sulfide with a 0.97:0.03 solid:liquid sulfide ratio. The modeled concentrations recreate measured concentrations after 1,300 ppm S (0.36 wt% FeS; dashed vertical line) is fractionated as sulfide. The shaded fields associated with the model curves show the uncertainty of the model propagated from the uncertainty of the applied sulfide-silicate melt partition coefficients[12], whereas those associated with the dashed lines showing metal concentrations in the SMI show the 1σ standard deviation of the measured values. In **a**, modeled Cu and Au concentrations (solid lines and fields) decrease from the initial values attained by 35% crystal fractionation until reaching the measured northern pSMI values (dashed lines and fields) as sulfide fractionation proceeds. In **b**, sulfide scavenging removes 60-70% of the initial Cu and Au but has little effect on the Ag budget. See the *Methods* for details of the estimation of the plausible extent of sulfide fractionation in the north.

References

1 Kay, S. M., Godoy, E. & Kurtz, A. Episodic arc migration, crustal thickening, subduction erosion, and magmatism in the south-central Andes. *Geological Society of America Bulletin* **117**, 67-88, doi:10.1130/b25431.1 (2005).

2 Hildreth, W. & Moorbath, S. Crustal contributions to arc magmatism in the Andes of central Chile. *Contrib. Mineral. Petrol.* **98**, 455-489, doi:10.1007/bf00372365 (1988).

3 Hickey-Vargas, R., Holbik, S., Tormey, D., Frey, F. A. & Moreno Roa, H. Basaltic rocks from the Andean Southern Volcanic Zone: Insights from the comparison of along-strike and small-scale geochemical variations and their sources. *Lithos* **258-259**, 115-132, doi:10.1016/j.lithos.2016.04.014 (2016).

4 Holm, P. M., Søager, N., Dyhr, C. T. & Nielsen, M. R. Enrichments of the mantle sources beneath the Southern Volcanic Zone (Andes) by fluids and melts derived from abraded upper continental crust. *Contributions to Mineralogy and Petrology* **167**, 1004, doi:10.1007/s00410-014-1004-8 (2014).

5 Stern, C. R. Subduction erosion: Rates, mechanisms, and its role in arc magmatism and the evolution of the continental crust and mantle. *Gondwana Research* **20**, 284-308, doi:10.1016/j.gr.2011.03.006 (2011).

6 Turner, S. J., Langmuir, C. H., Dungan, M. A. & Escrig, S. The importance of mantle wedge heterogeneity to subduction zone magmatism and the origin of EM1. *Earth and Planetary Science Letters* **472**, 216-228, doi:10.1016/j.epsl.2017.04.051 (2017).

7 Jacques, G. *et al.* Geochemical variations in the Central Southern Volcanic Zone, Chile (38–43°S): The role of fluids in generating arc magmas. *Chemical Geology* **371**, 27-45, doi:10.1016/j.chemgeo.2014.01.015 (2014).

8 Cembrano, J. & Lara, L. E. The link between volcanism and tectonics in the southern volcanic zone of the Chilean Andes: A review. *Tectonophysics* **471**, 96-113, doi:10.1016/j.tecto.2009.02.038 (2009).

9 Hickey-Vargas, R., Sun, M. & Holbik, S. Geochemistry of basalts from small eruptive centers near Villarrica stratovolcano, Chile: Evidence for lithospheric mantle components in continental arc magmas. *Geochimica et Cosmochimica Acta* **185**, 358-382, doi:10.1016/j.gca.2016.03.033 (2016).

10 Reubi, O. *et al.* Assimilation of the plutonic roots of the Andean arc controls variations in U-series disequilibria at Volcan Llaima, Chile. *Earth and Planetary Science Letters* **303**, 37-47, doi:10.1016/j.epsl.2010.12.018 (2011).

11 Reagan, M. K. *et al.* Time-scales of differentiation from mafic parents to rhyolite in North American continental arcs. *Journal of Petrology* **44**, 1703-1726 (2003).

12 Li, Y. & Audetat, A. Effects of temperature, silicate melt composition, and oxygen fugacity on the partitioning of V, Mn, Co, Ni, Cu, Zn, As, Mo, Ag, Sn, Sb, W, Au, Pb, and Bi between sulfide phases and silicate melt. *Geochim. Cosmochim. Acta* **162**, 25-45, doi:10.1016/j.gca.2015.04.036 (2015).

13 Jochum, K. P., Willbold, M., Raczek, I., Stoll, B. & Herwig, K. Chemical characterisation of the USGS Reference Glasses GSA-1G, GSC-1G, GSD-1G, GSE-1G, BCR-2G, BHVO-2G and BIR-1R using EPMA, ID-TIMS, ID-ICP-MS and LA-ICP-MS. *Geostandards and Geoanalytical Research* **29**, 285-302 (2005).

14 Jochum, K. P. *et al.* Determination of Reference Values for NIST SRM 610-617 Glasses Following ISO Guidelines. *Geostandards and Geoanalytical Research* **35**, 397-429, doi:10.1111/j.1751-908X.2011.00120.x (2011).

15 Fram, M. S. & Lesher, C. E. Geological constraints on mantle melting during creastion of the North Atlantic basin. *Nature* **363**, 712-714 (1993).
16 Li, C. & Ripley, E. M. Sulfur Contents at Sulfide-Liquid or Anhydrite Saturation in Silicate Melts: Empirical Equations and Example Applications. *Economic Geology* **104**, 405-412, doi:0361-0128/09/3818/405-8 (2009).
17 Palme, H. & O'Neill, H. S. C. in *Treatise on Geochemistry (Second Edition)* (eds Heinrich D. Holland & Karl K. Turekian) Ch. 3.1, 1-39 (Elsevier, 2014).
18 Salters, V. J. M. & Stracke, A. Composition of the depleted mantle. *Geochemistry, Geophysics, Geosystems* **5**, n/a-n/a, doi:10.1029/2003gc000597 (2004).
19 Sruoga, P., Llambías, E. J., Fauqué, L., Schonwandt, D. & Repol, D. G. Volcanological and geochemical evolution of the Diamante Caldera–Maipo volcano complex in the southern Andes of Argentina (34°10'S). *Journal of South American Earth Sciences* **19**, 399-414, doi:10.1016/j.jsames.2005.06.003 (2005).
20 Tormey, D. R., Frey, F. A. & Lopez escobar, L. Geochemistry of the active Azufre-Planchon-Peteroa volcanic complex, Chile (35o 15' S) - evidence for multiple sources and processes in a cordilleran arc magmatic system. *Journal of Petrology* **36**, 265-298, doi:10.1093/petrology/36.2.265 (1995).
21 Salas, P. A., Rabbia, O. M., Hernández, L. B. & Ruprecht, P. Mafic monogenetic vents at the Descabezado Grande volcanic field (35.5°S–70.8°W): the northernmost evidence of regional primitive volcanism in the Southern Volcanic Zone of Chile. *International Journal of Earth Sciences* **106**, 1107-1121, doi:10.1007/s00531-016-1357-5 (2017).
22 Whermann, H. *et al.* Insights from trace element geochemistry as to the roles of subduction zone geometry and subduction input on the chemistry of arc magmas. *International Journal of Earth Sciences* **103**, 1929-1944, doi:10.1007/s00531-013-0917-1) (2014).
23 Martínez, P., Singer, B. S., Roa, H. M. & Jicha, B. R. Volcanologic and petrologic evolution of Antuco-Sierra Velluda, Southern Andes, Chile. *Journal of Volcanology and Geothermal Research* **349**, 392-408, doi:10.1016/j.jvolgeores.2017.11.026 (2018).
24 Schindlbeck, J. C., Freundt, A. & Kutterolf, S. Major changes in the post-glacial evolution of magmatic compositions and pre-eruptive conditions of Llaima Volcano, Andean Southern Volcanic Zone, Chile. *Bulletin of Volcanology* **76**, doi:10.1007/s00445-014-0830-x (2014).
25 Hickey-Vargas, R., Moreno Roa, H., Lopez Escobar, L. & Frey, F. A. Geochemical variations in Andean basaltic and silicic lavas from from the Villarrica-Lanin volcanic chain (39.5° S): an evaluation of source heterogeneity, fractional crystallization and crustal assimilation. *Contributions to Mineralogy and Petrology* **103**, 361-386 (1989).
26 Turner, S. J., Langmuir, C. H., Katz, R. F., Dungan, M. A. & Escrig, S. Parental arc magma compositions dominantly controlled by mantle-wedge thermal structure. *Nature Geoscience* **9**, 772-776, doi:10.1038/ngeo2788 (2016).
27 Audétat, A., Dolejš, D. & Lowenstern, J. B. Molybdenite Saturation in Silicic Magmas: Occurrence and Petrological Implications. *Journal of Petrology* **52**, 891-904, doi:10.1093/petrology/egr008 (2011).
