# Supplementary material for: Sulfur and chlorine budgets control the ore fertility of arc magmas
Source: Nat Commun. 2022 Jul 21;13:4218. doi: 10.1038/s41467-022-31894-0 (PMC9304346; doi:10.1038/s41467-022-31894-0)
Supplement: Supplementary file 3 — Description of Additional Supplementary Files [file 41467_2022_31894_MOESM3_ESM.pdf]

Description of Additional Supplementary Files

File Name: Supplementary Data 1
Description: The major and trace element composition of the studied silicate melt inclusions and their host minerals, and the mineralogy and major and trace element composition of the studied rock samples.
